# Contemporary methods for the extraction and isolation of natural products

**DOI:** 10.1186/s13065-023-00960-z

**Published:** 2023-06-30

**Authors:** Milena Popova, Vassya Bankova

**Affiliations:** Institute of Organic Chemistry with Centre of Phytochemistry, Bulgarian Academy of Sciences, Acad. G. Bonchev str., bl. 9, Sofia, 1113 Bulgaria

## Abstract

Extraction is a vital step in obtaining pure bioactive natural compounds for medical, scientific and commercial use. Recently, interest in extracting natural products for applications across the food, pharmaceutical, and cosmetic industries has grown rapidly, driving demand for newer, more efficient extraction methods. To develop our understanding of this field, BMC *Chemistry* has launched a new article Collection titled “Contemporary methods for the extraction and isolation of natural products”.

Natural products have been used by humans for millennia for their therapeutic, cosmetic, and nutritional benefits. Today, these compounds continue to play a critical role in drug discovery and other industries. Extraction is the process of separating out the desired component(s) from a chemical mixture. It is a key step in using any bioactive natural compound. Advances in technology and research have led to the development of innovative approaches for extracting and isolating natural products. These contemporary methods have the potential to expand our understanding of natural product chemistry and unlock new sources of valuable compounds. In this collection, we aim to showcase the latest research and insights into contemporary methods for natural product extraction and isolation.

The first efforts by humans to make extractions from natural products probably coincided with the discovery of fire. Ancient cultures worldwide used extraction processes, primarily utilizing water as an extraction medium. Later on, distillation and alcohol extraction (preparation of tinctures) methods were adopted and refined [1].

The choice of solvent is the first important decision to be taken when designing an extraction procedure, as extraction solvents play a key role in determining the extracted compounds’ quality, quantity, and selectivity. The choice of extraction solvent depends on the chemical properties of the natural products being extracted and the desired end product. Solvents commonly used for natural product extraction include polar solvents such as water, methanol, ethanol, and acetone and non-polar solvents such as hexane, chloroform, and ethyl acetate. Each solvent has specific properties that make it suitable for extracting compounds of specific polarity. For example, ethanol–water mixtures are recommended for the extraction of phenolics [2]. However, acetone has been proven efficient in the extraction of polyphenols from lychee flowers compared to methanol, water and ethanol [3]. Solvents also play a role in the safety and potential environmental impact of the extraction process. It is important to consider the toxicity and flammability of solvents, as well as their potential effect on the environment. In this respect, organic solvents have a number of disadvantages: most of them are volatile and toxic and contribute significantly to environmental pollution [4]. With the development of green chemistry, the design of green and sustainable extraction methods of natural products has become a hot research topic. Among proposed green solvents, ionic liquids, natural deep eutectic solvents, supercritical and subcritical fluids and solvents from natural and renewable sources stand out as the most promising approaches for current solvent innovation [5–7].

The most common extraction method for natural products is typically solid-liquid extraction (the process of removing a solute component from the solid using a liquid solvent). Several solid-liquid extraction techniques have been applied to purify bioactive constituents from plants and microorganisms. Traditional methods include maceration, percolation, and Soxhlet extraction [8]. Recently developed extraction techniques for natural products are more energy efficient and result in shorter extraction times compared to earlier methods. Such techniques include ultrasound-assisted extraction, microwave-assisted extraction and pressurized solvent extraction. The choice of extraction solvent and method depends on the type of natural raw material being extracted, the target compounds, and the desired end product. Based on the law of similarity and intermiscibility (like dissolves like), solvents with a polarity value near the solute’s polarity are likely to perform better and vice versa. When selecting the extraction method, it is necessary to take into consideration the stability of the desired product: temperature should be chosen carefully in order to avoid degradation [9].

Contemporary methods for natural product extraction offer several benefits, including improved efficiency, reduced extraction time, and reduced solvent usage. However, there are also some challenges: Some methods, such as supercritical fluid extraction and microwave-assisted extraction, can be expensive due to the specialized equipment required to perform them. Given the costs involved, these same methods may also be challenging to scale up for commercial production. However, they also offer opportunities for increased productivity, potential selectivity, and sustainability. Therefore, researchers must carefully evaluate these methods to determine their suitability for specific applications and weigh the benefits against the costs and limitations.

Last but not least, interdisciplinary collaboration can contribute significantly to advancing this area of research. For example, a recent partnership of chemical engineers and biologists lead to clarification of the relations between extraction method and the antioxidant activity of C-phycocyanin [10]. The interest in the extraction and isolation of natural products and their various applications is growing, with the discovery of new and more diverse applications for natural compounds driving the development of more efficient extraction methods. We hope this development will continue at a pace inspired by global need and directed by the environmental, safety, and regulatory requirements such work demands.

## Data Availability

Not applicable.
